# Death of Wm. Weightman

**Published:** 1889-03

**Authors:** 


DEATH OF WM. WEIGHTMAN. Dr. Wm. Weightman, Jr., of the celebrated firm of Powers & Weightman, died after a brief illness on the morning of the 11th of Feb. He was the son of the original proprietor of this large establishment, and succeeded his father in the business. He was in the 46th year of his age.

